# Effect of Long-Term Potato Starch Retention with Citric Acid on Its Properties

**DOI:** 10.3390/molecules27082454

**Published:** 2022-04-11

**Authors:** Małgorzata Kapelko-Żeberska, Marta Meisel, Krzysztof Buksa, Artur Gryszkin, Antoni Szumny, Bogna Latacz, Bartosz Raszewski, Tomasz Zięba

**Affiliations:** 1 Department of Food Storage and Technology, Wroclaw University of Environmental and Life Sciences, Chełmońskiego 37, 51-630 Wrocław, Poland; marta.meisel@upwr.edu.pl (M.M.); artur.gryszkin@upwr.edu.pl (A.G.); bartosz.raszewski@upwr.edu.pl (B.R.); tomasz.zieba@upwr.edu.pl (T.Z.); 2 Department of Carbohydrates Technology, University of Agriculture in Krakow, Balicka 122, 30-149 Krakow, Poland; krzysztof.buksa@urk.edu.pl; 3 Department of Chemistry, Wroclaw University of Environmental and Life Sciences, C. K. Norwida 25, 50-375 Wrocław, Poland; antoni.szumny@upwr.edu.pl; 4 Institute of Sport, Tourism and Nutrition, Faculty of Biological Sciences, University of Zielona Gora, Licealna 9, 65-417 Zielona Gora, Poland; blatacz@uz.zgora.pl

**Keywords:** potato starch, citric acid, long-term retention, esterification, resistant starch

## Abstract

The present study aimed to determine changes in the properties of starch triggered by its long-lasting (1, 2, 4, 7, 10, or 14 days) retention with citric acid (5 g/100 g) at a temperature of 40 °C. The starch citrates obtained under laboratory conditions had a low degree of substitution, as confirmed via NMR and HPSEC analyses. The prolonging time of starch retention with citric acid at 40 °C contributed to its increased esterification degree (0.05–0.11 g/100 g), swelling power (30–38 g/g), and solubility in water (19–35%) as well as to decreased viscosity of the starch pastes. Starch heating with citric acid under the applied laboratory conditions did not affect the course of DSC thermal characteristics of starch pasting. The low-substituted starch citrates exhibited approximately 15% resistance to amylolysis.

## 1. Introduction

Starch and modified starch preparations are widely deployed in the industry. The most frequently applied methods of starch modification include the chemical ones. However, physical or enzymatic methods and their combinations also prove viable in this respect [1]. Starch esters produced via chemical starch modification have been described as highly applicable in the industry already in 1942 [2]. This finding has paved the way for a new trend of research into starch modification processes [3].

In recent years, there has been a growing interest in the use of citric acid. It is biodegradable and renewable, and also has been classified as safe for food uses because it is naturally derived via citrate fermentation. Starch can be esterified with citric acid in an aqueous suspension to produce low-substituted esters [4] or by roasting a dry mixture to produce esters with a high substitution degree dependent on process conditions [5]. Depending on esterification conditions and the degree of substitution of the resultant esters, the modified starch preparations can exhibit extremely different properties. Starch citrates may be both completely soluble and insoluble in water, susceptible or insusceptible to enzymatic degradation and pasting as well as form highly or low viscous pastes [6]. When starch is roasted with citric acid, two somewhat opposite processes take place. The first one involves acidic hydrolysis leading to the degradation of starch chains and, thus, to their increased solubility in water and susceptibility to amylolysis as well as to decreased viscosity of pastes formed. The second process entails starch esterification with citric acid, leading to its crosslinking and to the attachment of dextrins earlier detached during hydrolysis. The increased molecular weight of the ester triggers changes in the properties of the modified preparations “in the opposite trend” compared to those caused by hydrolysis. It is a dynamic system, dependent mainly on process temperature and duration, medium pH, and doses of reagents used. The ultimate properties of the modified preparation are a resultant of both these concomitant processes.

Starch esterification with citric acid is highly effective at high temperatures (120–150 °C), because these temperature conditions facilitate the formation an anhydride (highly reactive against starch) from citric acid in the first stage of the reaction [5]. The degree of substitution of the formed esters depends primarily on process temperature and concentrations of reagents [7]. The esterification reaction can also proceed at lower temperatures. Starch roasting at 100 °C produces a low-substituted ester exhibiting a significant solubility in water and swelling power [8,9]. Menzel et al. have demonstrated that starch was esterified even during five-hour drying of a starch mixture with citric acid at a temperature of 70 °C [10]. A question arises then: can the reaction of starch esterification with citric acid proceed at even lower temperatures? It is common knowledge that a temperature decrease of 10 °C causes a 2–3-fold decrease in the rate of a chemical reaction. Substantial elongation of the reaction may partly compensate for this process. Even minor starch esterification proceeding under these conditions upon citric acid coupled with the relatively weak hydrolytic effect of the acid on the polymer can produce a modified preparation with altered properties.

The present study aimed to determine changes in the properties of starch triggered by its long-lasting (1, 2, 4, 7, 10, or 14 days) retention with citric acid at a temperature of 40 °C, with special attention paid to the possibility of producing low-substituted starch citrates.

## 2. Discussion of Results

**Figure 1:**
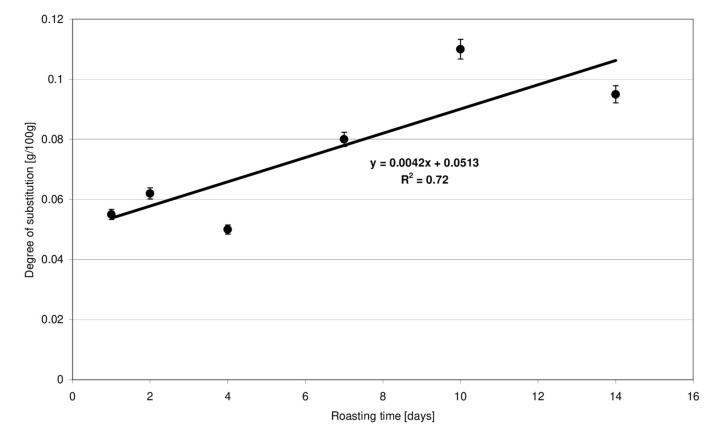
Effect of the time of starch retention with citric acid on the degree of substitution of modified starch preparations.

The ^1^HNMR technique is a highly reliable tool for ester bond detection in starch [11,12]. According to literature data [13], methylene protons of the unbound citric acid are visible as two doublets (2.75 and 2.65 ppm). In the starch citrate spectrum, these signals are shifted towards lower values: 2.54 and 2.48 ppm [14]. In the present study, the high intensity of chemical conversions proceeding at high temperatures was expected to be compensated for by a very long time of starch retention with citric acid at a relatively low temperature. Esters produced under these conditions had a very low degree of substitution. The NMR technique enabled only the qualitative determination of ester bonds featuring a characteristic signal shift to lower values. Due to the weak signals and, therefore, poorly readable NMR spectra, they were not included in the manuscript. A titration method was therefore employed for quantitative determination of the substitution degree. The usability of this method in respect of low-substituted citrates has been confirmed in the study by Menzel et al. [10]. The degree of esterification of starch preparations, expressed in grams of acid residues per 100 g of preparation dry matter, increased linearly accordingly to the determined function and ranged from 0.05 to 0.11 g (Figure 1). The degree of substitution of the preparations roasted with citric at for up to 4 days was, on average, two times lower compared to the preparations obtained after 10–14 days.

**Figure 2:**
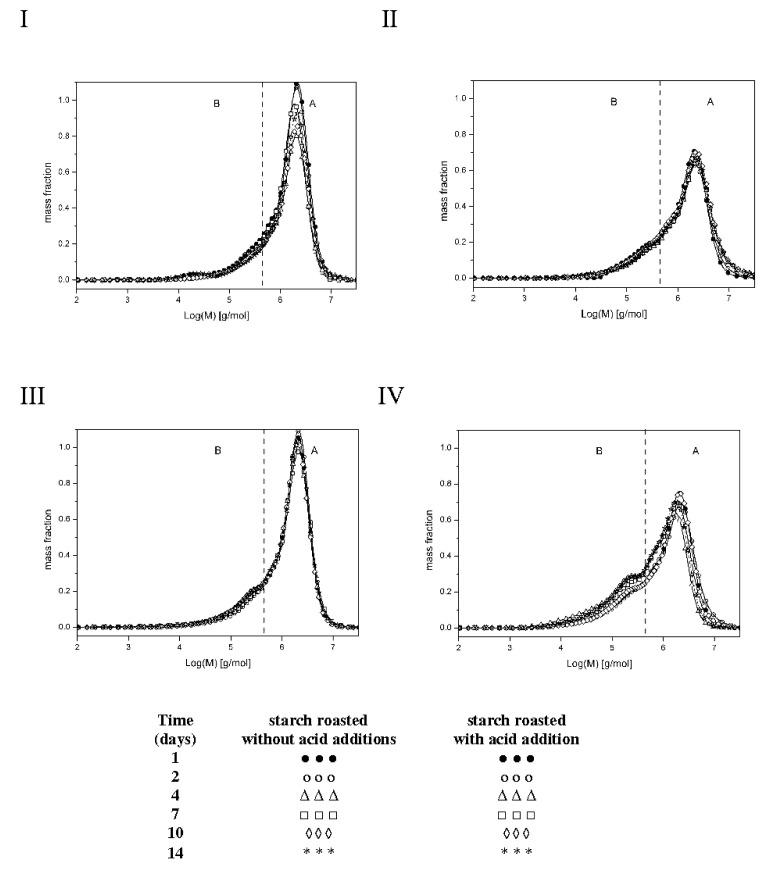
Molar mass distribution profiles of starches roasted without acid addition dissolved in DMSO (I), starches roasted without acid addition dissolved in NaOH (II), starches roasted with acid addition dissolved in DMSO (III), and starches roasted with acid addition dissolved in NaOH (IV).

The low substitution degree of starch raises some concerns over the methodological correctness of the experiment. Theoretically, there is a possibility of physicochemical inclusion of free acid in spiral coils of starch chains, which would however distort the result of esterification degree determination with a chemical method. A solution is found via the analysis of molar masses of preparations determined after dissolution in two different solvents: dimethylsulfoxide (DMSO) or an aqueous solution of sodium hydroxide (NaOH). DMSO is deemed to be a solvent, which causes no changes in the molar mass of starch dissolved in it. This finding is confirmed by the results obtained by various methods from, which are similar [8,14,15]. During starch dissolution, an aqueous NaOH solution triggers negligible hydrolytic changes [16]; however, due to the concomitant de-esterification process, this solvent is useful to assess the structure of starch esters [5]. Figure 2 shows the molar mass distribution profiles of the analyzed preparations, whereas Table 1 presents values of the weighted average of molar masses (Mw) of the preparations and dispersity (Ð = Mw/Mn; Mn—average molar mass) being a measure of the homogeneity of fractions constituting a given polymer [17]. The make the Ð value lower, the chromatograms were divided in fractions corresponding to high-molecular-weight (A) and low-molecular-weight (B) molecules.

Thermodynamic properties of aqueous solutions of maltodextrins from laser-light scattering, calorimetry and isopiestic investigations [17]. Relatively low values of Ð coefficient (1.4–5.7) point to insignificant differences in molar masses within particular starch fractions. The analysis of results obtained for the preparations dissolved in DMSO demonstrated no effect of the time of starch retention with citric acid on molar mass values (Mw), which ranged from 192.0 to 207.0 × 10^4^ g/mol for fraction A and from 20.3 to 22.4 × 10^4^ g/mol for fraction B and were similar to the Mw of the control sample (retained without the acid). Figure 2(I,III) indicate very similar molar mass distribution profiles of the analyzed samples. Dissolution of the control samples in the NaOH solution decreased its Mw by, on average, 8% in the case of fraction A and 11% on the case of fraction B. In turn, dissolution of starch retained with citric acid in the NaOH solution caused a significantly greater reduction in the mean molar masses, as evidenced in Figure 2(II,IV), i.e., 26% in the case of fraction A and 16% in the case of fraction B. Such a large decrease in Mw values caused by the NaOH solution is due not only to the hydrolytic effect of this solvent but also to the breakdown of crosslinking ester bonds. This corroborates the assumption that the long-term heating of starch with citric acid at a temperature of 40 °C caused its esterification. Presumably, starch retention with citric acid under the applied experimental conditions led to hydrolytic transformations, which were later somehow neutralized by ester crosslinking with citric acid. Similar conclusions were formulated by Kapelko et al., who proved that during starch roasting with citric acid, starch, and dextrins formed during concomitant acidic starch hydrolysis were easily ester-crosslinked [5].

**Figure 3:**
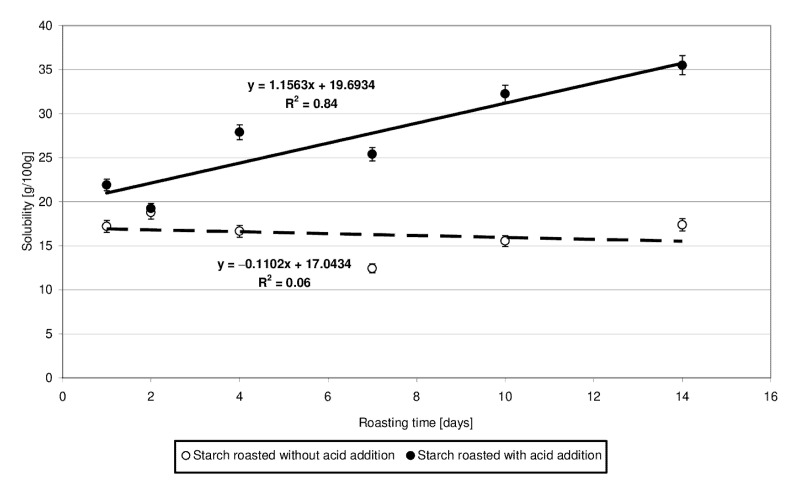
Dependency of solubility in water on the time of starch retention with and without citric acid.

Figure 3 presents the results of determinations of solubility of the produced starch preparations in water. Trend lines of changes in the analyzed starch property since its heating were plotted based on experimental data. The solubility of starch heated with citric acid was observed to increase accordingly with the determined linear function and was higher than the solubility of the samples roasted without acid addition. Based on the significant differences noted in the values of slope “a” and determination coefficient R^2^, it may be concluded that starch heating at 40 °C with citric acid had a several times stronger effect on its solubility in water than its heating without the acid. Presumably, the short-chain dextrins cleaved from amylopectin during acidic hydrolysis were highly soluble in water despite subsequent cross-linking with citric acid. Huang et al. [18] also have demonstrated that the water solubility of starch roasted with citric acid increased compared to that of the native starch. The increased solubility of starch heated with acid is due to acidic hydrolysis resulting in the synthesis of a high number of short-chain dextrins that easily dissociate and diffuse from starch granules during their swelling [18]. Olsson et al. [19] also demonstrated higher solubility of starch roasted with citric acid compared to native starch. It is worth emphasizing, however, that the solubility was higher in the case of starch heated at 70 °C compared to starch heated at temperatures exceeding 100 °C, which was due to the crosslinking reactions proceeding more intensively at the higher temperatures. The solubility of high-substituted starch citrates decreases along with an increasing citric acid addition [10].

**Figure 4:**
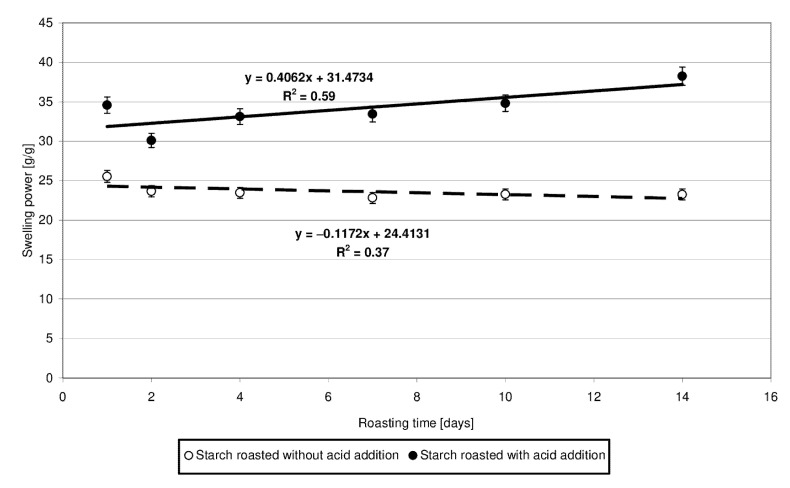
Dependency of swelling power on the time of starch retention with and without citric acid.

The swelling power of the starch heated without the acid approximated 23.5 g/g (Figure 4). Starch esters exhibited 7 to 15 g/g higher swelling power, and this difference was observed to increase along with prolonging time of starch retention, accordingly with the determined linear function. This increase is most likely due to the higher number of cross-linking bonds inside amylopectin, which increase its capability to absorb water. However, this is true only for the low-substituted esters. Zou et al. [20] demonstrated citric acid to act as a cross-linking agent, reducing the swelling power of the modified starch preparations. However, the low-substituted starch citrates produced at a temperature of 100 °C [5] or with the addition of only 5 g/100 g citric acid [21] were shown to exhibit a higher swelling power compared to the control samples. Increasing process temperature or acid addition level caused a significant decrease in the value of the analyzed traits, as confirmed elsewhere [22].

**Figure 5:**
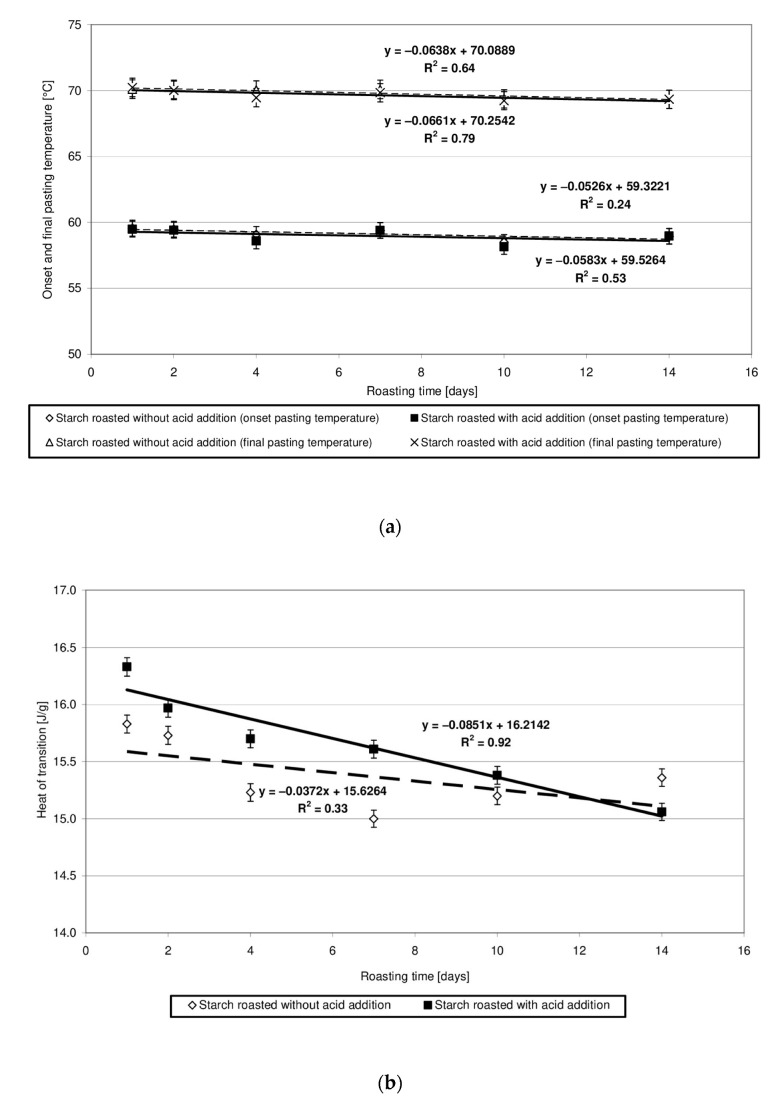
(**a**) Dependency of the onset and final pasting temperature on the time of starch retention with and without citric acid. (**b**) Dependency of the heat of transition on the time of starch retention with and without citric acid.

Potato starch forms pastes in a temperature range of 60 to 70 °C, and the heat of this transition approximates 17 J/g [23]. The heat of transition reflects the energy required to disturb the granular structure and is associated with the total crystallinity of starch granules [24]. Changes in the values of parameters read out from the pasting characteristics of starch heated at 40 °C without and with the addition of citric acid are similar and show a slightly downward trend (Figure 5a,b). This finding indicates similar, negligible changes in the physical structure of both native starch and produced starch esters. Due to the very high number of thermograms sharing a similar course, they were not presented in the manuscript. In addition, other researchers [24] reported on the lack of changes in the pasting characteristics of low-substituted starch citrates. A significant decrease in the heat and temperature of pasting [7,21], ending ultimately in the starch’s inability to form pastes [5], have already been observed along with an increased degree of starch citrate substitution.

**Figure 6:**
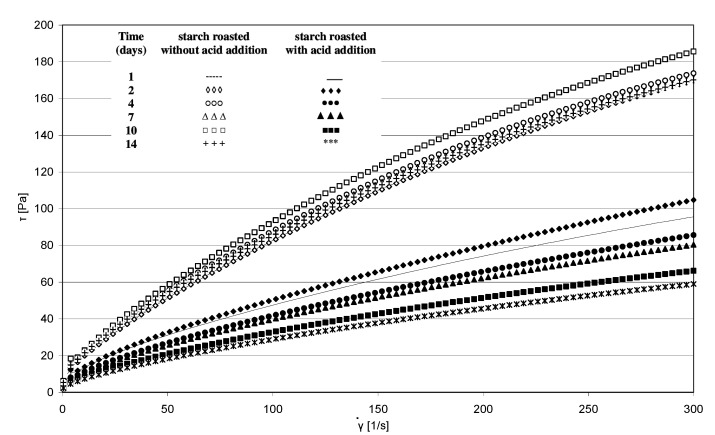
Flow curves of pastes produced from starch retained for various periods of time with and without citric acid.

**Figure 7:**
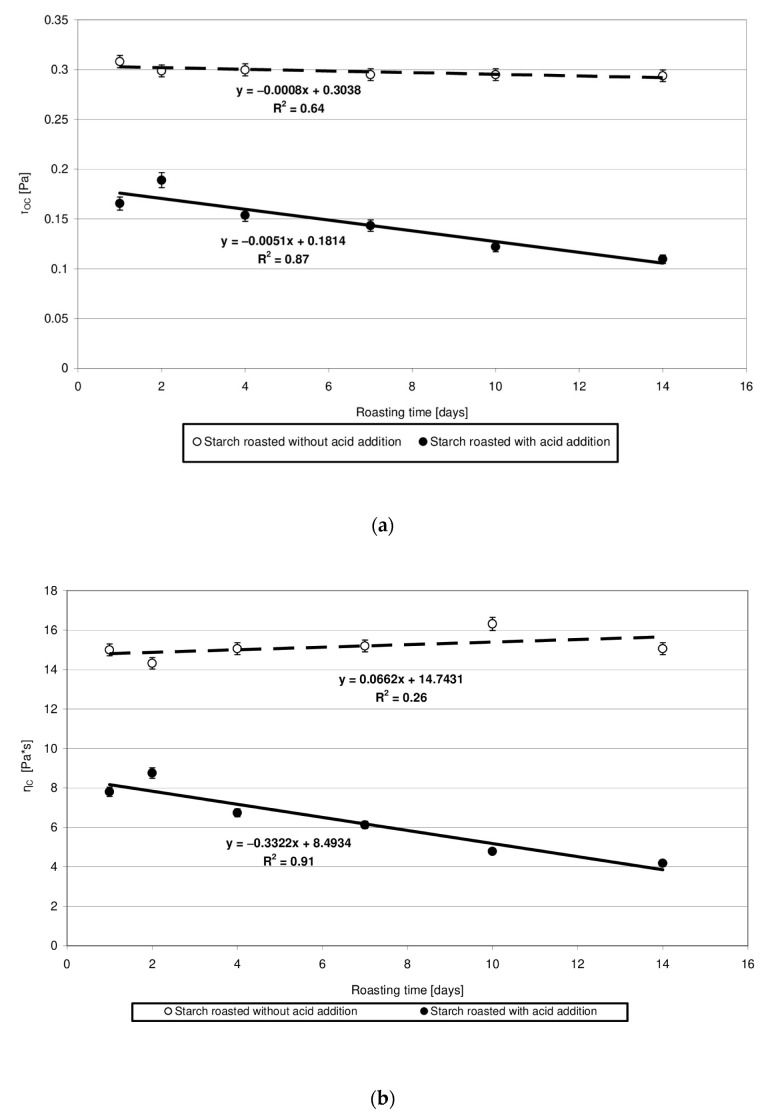
(**a**) Dependency of the Casson’s coefficient (τ_oc_) on the time of starch retention with and without citric acid. (**b**) Dependency of the Casson’s plastic viscosity (η_C_) on the time of starch retention with and without citric acid.

The rheological properties of pastes prepared from the analyzed starches were determined based on the plotted flow curves (Figure 6). The course of these curves was typical of starch pastes, i.e., shear-thinned non-Newtonian fluids. A similar course of starch paste flow curves was reported by other authors [25,26]. Starch retention for 14 days at a temperature of 40 °C without acid addition caused no significant changes in the rheological properties of pastes made of it. This is indicated by the similar course of flow curves and similar values of rheological coefficients (Figure 7a,b). The multi-fold increase in the values of the determination coefficient *R*^2^ and absolute slope “a”, observed in the case of analogous trend lines determined for starch samples heated with citric acid, points to the effect of this factor on paste rheology. The viscosity of pastes in the entire course of flow curves decreased along with extended starch heating with citric acid. A similar tendency was observed in the case of the rheological coefficients. The analysis of the occurring changes may lead to false conclusions that the paste viscosity decrease is attributable only to the acid effect on starch. However, the above-described chromatographic analysis indicates that there was no decrease in the molar mass of both types of starches (heated with and without acid addition) after their dissolution in DMSO. This decrease occurred only after starch dissolution and esterification in the NaOH solution, which proves the hydrolytic and, simultaneously, the crosslinking effect of citric acid on starch. Therefore, it can be concluded that the viscosity decrease was not due to the reduction in molar mass but rather to changes in the spatial arrangement of starch chains. The effect of the spatial arrangement of molecules on the properties of the analyzed substance is a well-known phenomenon [15].

**Figure 8:**
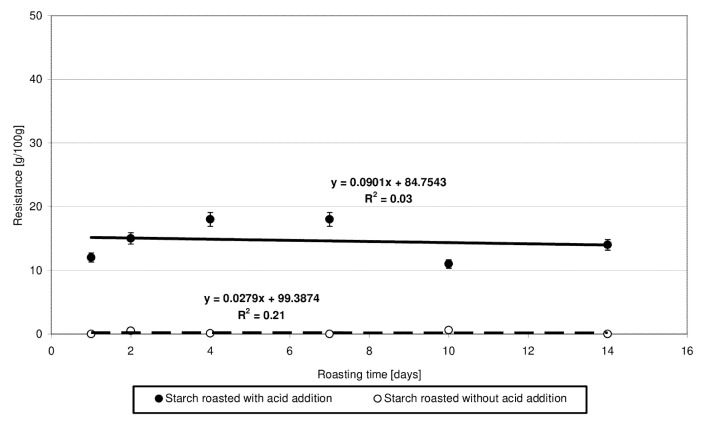
Dependency of resistance on the time of starch retention with and without citric acid.

Starch susceptibility to enzymatic hydrolysis is determined mainly by its botanical origin [18,27] and by its physical [18,27] or chemical [8,18,27] modification. The low-substituted starch citrates obtained in the present study exhibited a several-percentage resistance to amylolysis (Figure 8), which was not affected by the time of starch heating with citric acid and, consequently, by the degree of ester substitution. The very fact of change in the resistance of starch heated with citric acid compared to native starch is another proof of esterification taking place under the applied experimental conditions because it is the chemical modification that hinders the access of enzymes to the hydrolyzed starch chains. Similar conclusions have been formulated by other authors [8,18]. As evidenced in other research, the resistance to amylolysis depends not only on the degree of esterification but also on the conditions of this reaction or the substitution site of acid residues in a glucose chain [5].

## 3. Materials and Methods

### 3.1. Materials

The research material included Superior Standard potato starch produced in 2019 at the Przedsiębiorstwo Przemysłu Spożywczego PEEPES in Łomża (Poland). Starch was modified with citric acid purchased at POCH SA Gliwice (Gliwice, Poland).

### 3.2. Production of Modified Starch Preparations

Citric acid was dissolved in water, and then solution pH was brought to pH = 3.5 using 10 M NaOH solution. The solution was mixed with starch used in a dose to ensure acid content in the mixture at 5 g/100 g after drying. A moist mixture was carefully mashed through a screen, mixed, and conditioned for 12 h at room temperature. Afterward, it was divided into 6 portions that were left on trays in a drying cabined (Memmert, Germany) at a temperature of 40 °C for 1, 2, 4, 7, 10, or 14 days. Each sample was rinsed six times with 65% ethyl alcohol. The produced preparations were dried in an air dryer (Memmert, Germany) at a temperature of 35 °C for 12 h, ground in a laboratory grinder, and sieved through a screen with mesh size of 400 μm.

The preparations produced analogously as the esterified preparations but without the addition of citric acid served as the control.

### 3.3. Determination of the Degree of Substitution 

Two grams of the analyzed preparation were weighed exactly to 0.001 g and mixed with 2 mL of distilled water and 50 mL of 1 N KOH solution. The mixture was heated in a boiling water bath for 10 min. After cooling, it was neutralized to pH = 8.5 using 5 N acetic acid solution. Then, 25 mL of borate buffer (pH = 8.5) and 0.3 g of an indicator (a mixture of murexide and Na_2_SO_4_, 1:500) were added. The mixture was filled with water to the volume of 300 mL and titrated with 0.05 M CuSO_4_ solution until its red-purple color disappeared. The degree of starch esterification with citric acid was expressed in grams of citric acid residues per 100 g of the preparation [28].

### 3.4. Determination of the Degree of Substitution (DS) with the Technique of Nuclear Magnetic Resonance ^1^H NMR 

This analysis was carried out the Laboratory of Structural NMR Analyses at the Chemical Faculty of the Wrocław Technical University (Wrocław, Poland) using a Bruker Avance II 600 MHz spectrometer. The analyzed starch preparations were dissolved in DMSO-d6 (20 mg in 0.6 mL). The analysis was conducted at a temperature of 25 °C [5].

### 3.5. Molar Mass (Mw) Analysis 

Distribution of molar masses of the examined starches was evaluated by modified HPSEC/RI methods Buksa et al. and Praznik et al. [29,30]. Prior to injection, 20 mg of the sample were dissolved in (a) 6 mL of DMSO at 70 °C, for 24 h; (b) 4 mL of 0.5 M NaOH at 35 °C for 24 h using a magnetic stirrer. After dissolving, the solution (b) was neutralized with 2 mL of 1 M HCl. Afterward, the solutions (a) or (b) were centrifuged for 5 min at 2000× *g*, and the supernatant was injected into the columns. The system consisted of a series of columns: OHpak SB-G (guard), OHpak SB-806, and OHpak SB-804 Shodex (Japan). An aqueous solution of 100 mM NaNO_3_ was used as an eluent. Flow rate was 0.6 mL min^–1^, and the injection loop was 100 mL. The temperature of columns was set at 60 °C. The molar mass distribution of the starch was measured using refractive index (RI) detection. A calibration curve was plotted with pullulan standards (Shodex Standard, Macherey–Nagel) with known molecular masses (P-5, 10, 100, 400, and 800) and glucose. Each standard (10 mg) was dissolved for 1 h in the eluent (4 mL) and measured in the same way as the samples. Molar mass distribution was used to calculate the molecular parameters: average molar mass (Mw, Mn) and dispersity (Ð = Mw/Mn) using Eurochrom (ver. 3.05, Knauer, Berlin, Germany) and Clarity (ver. 4.0.1.700, DataApex, Prague, Czech Republic) software.

The molar mass distribution profiles were divided into 3 fractions: fraction “A” of Mw > 40.3 × 10^4^ g/mol, fraction “B” of 40.3 × 10^4^ > Mw > 0.6 × 10^4^ g/mol, and fraction “C” of Mw < 0.6 × 10^4^ g/mol, and molecular parameters of each fraction were calculated.

### 3.6. Swelling Power and Solubility of Starch Preparations in Water Having a Temperature of 80 °C 

In brief, 200 mL of an aqueous suspension containing 1 g of starch preparations or modified starch preparations per 100 g solution were prepared in a round-bottom flask. The flask was placed in a water bath with shaking at a temperature of 80 °C. It was kept under these conditions for 30 min until the water bath temperature had been obtained inside it. Afterward, the flask was cooled to a temperature of 20 °C, and water evaporated during heating was supplemented. Next, 50 g of the starch suspension were weighed into centrifuge tubes, which were then centrifuged in a Biofuge 28RS Heraeus Sepatech centrifuge at 14.500 rpm and 20 °C for 30 min. Next, the supernatant was decanted, and its dry matter content was determined with the air-dry method at a temperature of 105 °C. The precipitate left in the tubes was weighed [31].

### 3.7. Determination of the Characteristics of Phase Transitions of Starch Preparations with Differential Scanning Calorimetry (DSC) 

This determination was conducted using a DSC 822E differential scanning calorimeter (Mettler Toledo, Brno, Czech Republik) [32]. Pre-tests were performed in a temperature range of 25–100 °C. Aluminum crucibles (100 μL) with lids were used for analyses. A 10 mg starch sample (per starch dry matter) was weighed and placed on crucible’s bottom, then distilled water was added in the ratio of 3:1 respective to sample weight. The measuring crucible was covered with a lid, conditioned at 20 °C for 30 min. Next, it was transferred to an oven chamber having a temperature of 25 °C, and heated to a temperature of 100 °C at the heating rate of 4 °C/min.

### 3.8. Rheological Properties of Starch Pastes 

Analyses were carried out using an RS 6000 oscillating-rotating viscosimeter (Haake, Karlsruhe, Germany) for starch suspensions contained 5 g of starch preparations or modified starch preparations per 100 g solution that were heated at 96 °C for 30 min under continuous stirring. The properties of the prepared pastes were determined based on the flow curves [33].

The flow curves were plotted for the pastes at a measurement temperature of 50 °C and a shear rate of 1–300 s^−1^. A hot paste (38 g) was placed in a system of coaxial cylinders (Z38AL type) of an RS 6000 rheometer, then cooled, and relaxed at the measurement temperature for 15 min. The flow curves plotted were described using the following equations:

Casson: *τ*^0.5^ = *τ_oc_^0^*^.5^ + (*ƞ_c_*·*ẏ*)^0.5^

where: *τ*—shear stress (Pa), *y*—shear rate (s-1), *τ_oc_*—yield point (Pa), *η_c_*—Casson’s plastic viscosity (Pa s).

### 3.9. Resistance of Starch Preparations to the Action of Amyloglucosidase 

A 0.36 g portion of starch preparations or modified starch preparations per 100 g solution was prepared in a conical flask, which was then kept at a boiling temperature for 5 min. The suspension was then cooled, evaporated water was completed to the weight of 38 g, and 34 mL of an acetate buffer (pH 4.5) were added. The flask was placed in a water bath with a shaker at a temperature of 37 °C, and 4 mL of an amyloglucosidase solution was added (enzyme to acetate buffer ratio was 1:100). After 20 min, and 1, 2, or 3 h (or till the maximal saccharification of the preparation), 1 mL of the hydrolysate was collected to a centrifuge tube and centrifuged at 5000 rpm for 5 min in an MPW 312 centrifuge. Then, 10 μL of the supernatant were collected from the centrifuge sample, transferred to a microcuvette containing 1 mL of a BIOSYSTEM reagent, stirred, and incubated at a temperature of 20 °C for 15 min. Absorbance was measured at a wavelength of λ = 500 nm using a CECIL CE 2010 colorimeter against a blank sample made of the reagent with the acetate buffer. The content of glucose was read out from the standard curve [34].

### 3.10. Statistical Analysis

Experimental results were subjected to a statistical analysis using Statistica 13.0 package (StatSoft, Cary, North Carolina, USA). The statistical computations (from at least three parallel replications) enabled determining values of the least significant differences (LSD) and standard deviations.

The experimental data achieved were then used to determine correlations between selected traits that were described with linear (second order polynomial) equations.

## 4. Conclusions

Starch heating with citric acid resulted in the production of low-substituted esters, whose degree of substitution ranged from 0.05 to 0.11 g of acid residues per 100 g of starch. The produced esters exhibited increased resistance (by approximately 15%) to amylolysis, being typical of RS4 type resistant starch. The comparison of molar mass distribution of these esters with control samples proves the existence of cross-linking bonds in the modified preparations. The prolonging time of starch retention with citric acid at 40 °C contributed to its increased esterification degree, swelling power, and solubility in water as well as to decreased viscosity of the starch pastes. Starch retention with citric acid under laboratory conditions did not affect the course of DSC thermal characteristics of starch pasting.

## Figures and Tables

**Table 1:** Weighted average of molar masses and molar dispersity of starches retained for various periods of time with or without citric acid after dissolution in DMSO or NaOH.

Time [Days]	Fraction	Solvent Type							
		DMSO				NaOH			
		Preparation Type							
		Without Acid		With Acid		Without Acid		With Acid	
		M_w_ × 10^4^ [g/mol]	Đ	M_w_ × 10^4^ [g/mol]	Đ	M_w_ × 10^4^ [g/mol]	Đ	M_w_ × 10^4^ [g/mol]	Đ
1	A	209.8	1.5	194.3	1.5	152.6	1.5	156.0	1.5
	B	20.9	5.7	20.5	3.3	19.4	2.2	18.0	2.6
2	A	203.8	1.5	200.4	1.5	168.2	1.5	170.3	1.6
	B	21.4	2.7	21.9	2.1	19.4	2.2	18.4	2.6
4	A	183.2	1.4	207.0	1.5	193.0	1.7	137.8	1.4
	B	22.4	3.7	20.4	5.5	19.2	2.9	17.6	2.8
7	A	216.0	1.5	197.3	1.5	208.1	1.8	127.3	1.4
	B	20.3	3.0	20.6	2.7	18.1	2.4	16.6	2.4
10	A	212.5	1.5	192.0	1.5	204.1	1.8	156.0	1.5
	B	21.9	2.2	20.9	2.1	19.1	2.2	18.0	2.6
14	A	193.9	1.5	194.9	1.5	195.6	1.7	132.0	1.5
	B	22.2	2.7	21.1	2.2	19.1	3.2	16.3	3.6

## Data Availability

Data are contained within the article.

## Sample Availability

Samples of the compounds are not available from the authors.
